# In-vitro and in-vivo phenotype of type Asia 1 foot-and-mouth disease viruses utilizing two non-RGD receptor recognition sites

**DOI:** 10.1186/1471-2180-11-154

**Published:** 2011-06-29

**Authors:** Pinghua Li, Zengjun Lu, Huifang Bao, Dong Li, Donald P King, Pu Sun, Xingwen Bai, Weijun Cao, Simon Gubbins, Yingli Chen, Baoxia Xie, Jianhong Guo, Hong Yin, Zaixin Liu

**Affiliations:** 1 State Key Laboratory of Veterinary Etiological Biology, National Foot and Mouth Disease Reference Laboratory, Key laboratory of Animal Virology of Ministry of Agriculture, Lanzhou Veterinary Research Institute, Chinese Academy of Agricultural Sciences, No. 1 Xujiaping, Yanchangbao, Lanzhou, Gansu 730046, PR China; 2 Institute for Animal Health, Pirbright Laboratory, Ash Road, Pirbright, Woking, Surrey, England GU24 0NF, UK

## Abstract

**Background:**

Foot-and-mouth disease virus (FMDV) uses a highly conserved Arg-Gly-Asp (RGD) triplet for attachment to host cells and this motif is believed to be essential for virus viability. Previous sequence analyses of the 1D-encoding region of an FMDV field isolate (Asia1/JS/CHA/05) and its two derivatives indicated that two viruses, which contained an Arg-Asp-Asp (RDD) or an Arg-Ser-Asp (RSD) triplet instead of the RGD integrin recognition motif, were generated serendipitously upon short-term evolution of field isolate in different biological environments. To examine the influence of single amino acid substitutions in the receptor binding site of the RDD-containing FMD viral genome on virus viability and the ability of non-RGD FMDVs to cause disease in susceptible animals, we constructed an RDD-containing FMDV full-length cDNA clone and derived mutant molecules with RGD or RSD receptor recognition motifs. Following transfection of BSR cells with the full-length genome plasmids, the genetically engineered viruses were examined for their infectious potential in cell culture and susceptible animals.

**Results:**

Amino acid sequence analysis of the 1D-coding region of different derivatives derived from the Asia1/JS/CHA/05 field isolate revealed that the RDD mutants became dominant or achieved population equilibrium with coexistence of the RGD and RSD subpopulations at an early phase of type Asia1 FMDV quasispecies evolution. Furthermore, the RDD and RSD sequences remained genetically stable for at least 20 passages. Using reverse genetics, the RDD-, RSD-, and RGD-containing FMD viruses were rescued from full-length cDNA clones, and single amino acid substitution in RDD-containing FMD viral genome did not affect virus viability. The genetically engineered viruses replicated stably in BHK-21 cells and had similar growth properties to the parental virus. The RDD parental virus and two non-RGD recombinant viruses were virulent to pigs and bovines that developed typical clinical disease and viremia.

**Conclusions:**

FMDV quasispecies evolving in a different biological environment gained the capability of selecting different receptor recognition site. The RDD-containing FMD viral genome can accommodate substitutions in the receptor binding site without additional changes in the capsid. The viruses expressing non-RGD receptor binding sites can replicate stably in vitro and produce typical FMD clinical disease in susceptible animals.

## Background

Foot-and-mouth disease virus (FMDV) is an important animal pathogen that causes a severe vesicular disease in cattle, swine, sheep and other cloven-hoofed animals [1,2]. The virus belongs to the Aphthovirus genus within the *Picornaviridae *family [3]. The genome is a positive-sense single-stranded RNA molecule that is encapsidated by 60 copies of each of the four structural polypeptides of which VP4 is internal and the others (VP1, VP2 and VP3) are exposed [4]. It has been shown that VP1 is the most variable among the capsid polypeptides, and it is widely used to characterize field strains of FMDV to provide data to support epidemiological investigations of disease outbreaks among livestock.

A major, highly variable antigenic site of FMDV is located at the exposed G-H loop comprising amino acids 134-160 of the capsid protein VP1 [4-6], which plays an important role in cell infection and is also a major target for protective host responses mediated via humoral immunity [5,7-9]. This mobile loop contains a conserved Arg-Gly-Asp (RGD) motif that has been shown to be a major determinant in the interaction of the virus with cell surface receptors of the integrin superfamily [7,10,11]. Indeed, previous studies, using different approaches, have indicated that naturally occurring field isolates of FMDV bind to cells via these highly conserved surface-exposed RGD residues [11,12]. In particular, it has been reported that FMD viruses utilize multiple RGD-dependent integrins of the αv subgroup to initiate infection, including αvβ3, αvβ6, αvβ1 and αvβ8 [13-17]. However, the RGD integrin recognition domain can become dispensable upon in-vitro passage of FMDV: multiple phenotypic changes that are associated with a limited number of amino acid substitutions at the capsid surface which may even include modifications within the RGD triplet [18-21]. Tissue culture-adapted viruses can use heparan sulfate (HS) as a receptor to enter cells [18,22], and can also dispense with their RGD integrin-binding motif [23]. These findings indicate the existence of alternative RGD-independent pathways for FMDV entry into cell.

In the present study we report that two viruses harboring alternative receptor binding sites (RDD or RSD) were generated after short-term passage of an FMDV field isolate (Asia1/JS/CHA/05) in different environments. The non-RGD receptor recognition motifs were stably maintained during subsequent passage in cell culture. To study the ability of an RDD-containing FMD viral genome to accommodate substitution in receptor binding site and non-RGD viruses to cause disease in susceptible animals, we assembled an RDD-containing FMDV (Asia1/JSp1c8) full-length cDNA clone and derived mutant clones harboring RGD or RSD motif with a single amino acid substitution (RDD→RGD, RDD→RSD) in the receptor binding site. Following transfection of BSR/T7 cell with three full-length plasmids, the resulting viruses were examined for their infectious potential in-vitro and in-vivo.

## Results

### Sequence analysis of Asia1/JS/CHA/05 and its derivatives

Deduced amino acid sequence analysis of the 1D-encoding region showed that Asia1/JS/CHA/05 had a consensus RGD triplet at position 143-145 of VP1, while Asia1/JSp1c8 obtained an alternative RDD triplet at this position. However, careful inspection of the electropherograms from the Asia1/JSM4 VP1 gene sequencing reactions revealed the presence of two genetic subpopulations, one with RGD and the other with RSD at receptor binding site. To further investigate the genetic heterogeneity within these samples, 10 biological clones containing VP1 genes of each Asia1/JS/CHA/05, Asia1/JSp1c8 and Asia1/JSM4 were sequenced. The 10 clones obtained from each of the Asia1/JS/CHA/05 and Asia1/JSp1c8 viruses respectively encode RGD and RDD tripeptide at position 143-145 of VP1. For Asia1/JSM4, four clones encoded RSD and six clones maintain the RGD motif at the same position. These results were identical to the amino acid sequence analysis performed by direct sequencing of PCR amplicons. Additionally, amino acid sequence analysis of the capsid-coding regions of Asia1/JS/CHA/05, Asia1/JSp1c8, and Asia1/JSM4 showed that Asia1/JSp1c8 had seven amino acid substitutions in the capsid region (1 in 1A, 3 in 1B, 1 in 1C and 3 in 1D; Table 1) compared with Asia1/JS/CHA/05 and Asia1/JSM4.

**Table 1:** Comparison of the P1 amino acid sequence of Asia1/JS/CHA/05, Asia1/JS/p1c8, and Asia1/JSM4

Capsid region	**Amino acid residue position**^***a***^	Asia1/JS/CHA/05	Asia1/JSM4	Asia1/JS/p1c8
1A	73	S	S	N
1B	107	I	I	V
	132	E	E	K
	134	D	D	G
1C	133	T	T	A
1D	144	G	G/S	D
	154	N	N	S
	202	K	K	E

^*a *^Amino acid residues are numbered from the amino terminus to the carboxyl terminus. Single letter amino acid code is used.

**Figure 1:**
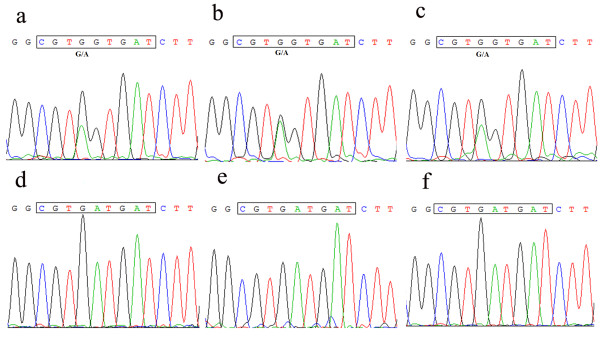
**Sequencing electropherograms of the VP1 PCR-amplicons of derivatives derived from Asia1/JSM4 and Asia1/JSp1c8**. The nucleotides encoding receptor-binding tripeptide are boxed, (a, b, c) represent sequencing electropherograms of Asia1/JSM6c5, Asia1/JSM6c15, and Asia1/JSM6c20, respectively; (c, d, e) represent sequencing electropherograms of Asia1/JSp1c8, Asia1/JSp1c15, and Asia1/JSp1c20, respectively.

Taken together, these results suggested that the RDD mutants became dominant or obtained equilibrium with the RGD and RSD subpopulations at an early phase of Asia1/JS/CHA/05 quasispecies evolution. Since RDD and RSD motifs are unusual in lacking the RGD integrin-recognition sequence, additional multiple passages were performed to determine its stability. Amino acid sequence of the VP1 gene of the viruses obtained from different passages of Asia1/JSp1c8 and Asia1/JSM4 revealed that the RDD and RSD sequence were genetically stable for at least 20 passages (Figure 1). The amino acid sequences of the G-H loop of viruses derived from different passages are summarized in table 2. Evidence that FMDVs can contain an RDD or RSD receptor-binding site increases the quasispecies complexity around the RGD-coding region.

**Table 2:** Comparison of amino acid sequence at G-H loop of VP1 of the viruses derived from different origins and full-length plasmids

Virus/plasmid	**Encoded G-H loop amino acid sequence**^***c***^	Additional amino acid changes in VP1
Asia1/JS/CHA/05	TTYGEESSR**RGD**LAALARRVNNRLPTS	-

Asia1/JSp1	TTYGEESSR**RGD**LAALARRVNNRLPTS	-
Asia1/JSp1c4	TTYGEESSR**RDD**LAALARRVSNRLPTS	N154S
Asia1/JSp1c8	TTYGEESSR**RDD**LAALARRVSNRLPTS	N154S
Asia1/JSp1c20	TTYGEESSR**RDD**LAALARRVSNRLPTS	N154S
Asia1/JSM4	TTYGEESSR**RGD**LAALARRVNNRLPTS	-
	TTYGEESSR**RSD**LAALARRVNNRLPTS	-
Asia1/JSM6c20	TTYGEESSR**RGD**LAALARRVNNRLPTS	-
	TTYGEESSR**RSD**LAALARRVNNRLPTS	-
pRDD	TTYGEESSR**RDD**LAALARRVSNRLPTS	-
pRSD	TTYGEESSR**RSD**LAALARRVSNRLPTS	-
pRGD	TTYGEESSR**RGD**LAALARRVSNRLPTS	-
FMDV-RDD^a^	TTYGEESSR**RDD**FAALARRVSNRLPTS	L146F
FMDV-RSD^a^	TTYGEESSR**RSD**LAALARRVSNRLPTS	N154S
FMDV-RGD^a^	TTYGEESSR**RGD**FAALARRVSNRLPTS	L146F
FMDV-RDD/pig^b^	TTYGEESSR**RDD**LAALARRVSNRLPTS	-
FMFV-RDD/bovine^b^	TTYGEESSR**RDD**LAALARRVSNRLPTS	-
FMDV-RSD/pig^b^	TTYGEESSR**RSD**LAALARRVSNRLPTS	-
FMDV-RSD/bovine^b^	TTYGEESSR**RSD**LAALARRVSNRLPTS	-

^*a *^The rescued viruses were passaged 20 times in cell culture.

^*b *^Virus recovered from vesicular lesions, away from the inoculation site.

^*c *^Sequence data were obtained by RT-PCR of the VP1 capsid region. The dashes represent receptor binding triplet of the viruses derived from different origins and full-length plasmids.

### Rescue of viable viruses from the full-length cDNA clones

To examine the influence of single amino acid substitutions in the receptor binding site of the RDD-containing FMD viral genome on virus viability and the ability of non-RGD viruses to cause disease in susceptible animals, we assembled a full-length cDNA clone of an RDD-containing FMDV and derived mutant clones containing RSD or RGD motifs with a single amino acid substitution in the receptor binding site (RDD→RGD, RDD→RSD). BSR-T7/5 cells were independently transfected with linearized-plasmids, pRDD, pRGD and pRSD. The typical FMDV cytopathic effect was clearly visible at 36 h post-transfection with pRDD and pRGD, and at 48 h post-transfection with pRSD. The three rescued viruses were named FMDV-RDD, FMDV-RGD, and FMDV-RSD, respectively.

**Figure 2:**
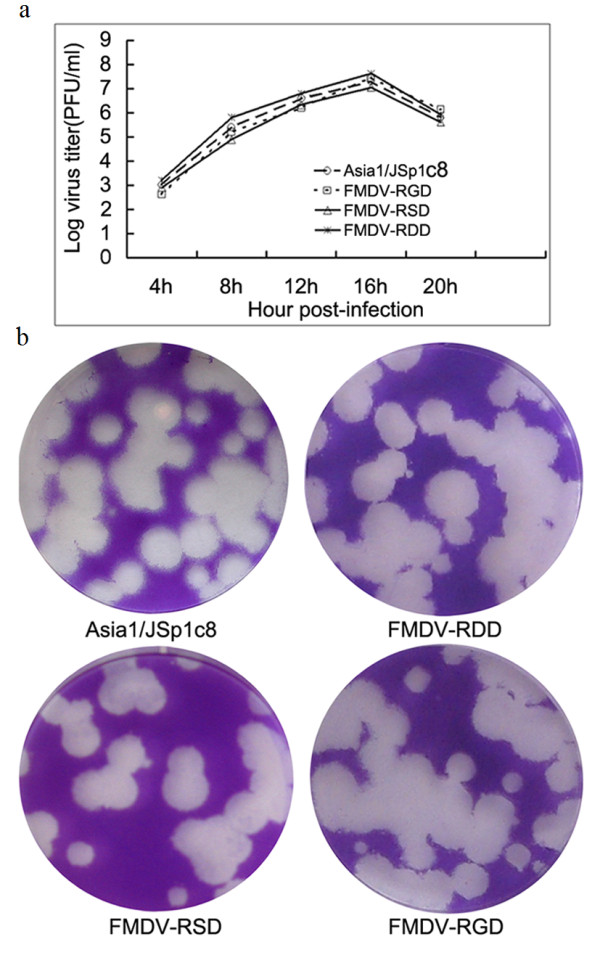
**Growth characteristics of three rescued viruses in cell culture compared with parental virus**. (a), One-step growth curves of the parental and three cloned viruses. (b), Morphology of plaques formed in BHK-21 cell monolayers by the parental and three cloned viruses.

To increase the virus titers, all rescued viruses were subjected to serial passage in BHK-21 cells, after which the VP1 sequence was analyzed to confirm that the recovered viruses had maintained the cDNA-encoded receptor binding motifs (Table 2). When the growth characteristics of the rescued viruses were compared with the parental virus Asia1/JSp1c8 by one-step growth kinetics assays, rescued viruses showed similar growth properties to the parental virus (Figure 2a). In addition, the plaque sizes of the parental virus and the rescued viruses were also similar (Figure 2b). These results suggest that single amino acid substitutions in the receptor binding site of Asia1/JSp1c8 virus do not affect virus viability.

### The pathogenicity of the rescued viruses in cattle and swine

**Figure 3:**
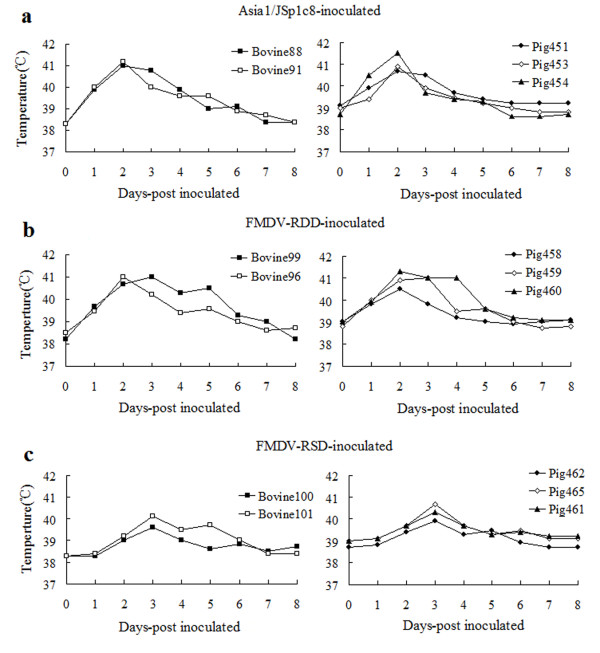
**Rectal temperatures of all FMDV inoculated animals**. (a), Temperatures in Asia1/JSp1c8-inoculated animals; (b), Temperatures in FMDV-RDD-inoculated animals; (c), Temperatures in FMDV-RSD-inoculated animals.

To investigate the pathogenicity of the non-RGD viruses in the natural host, we performed direct inoculation of parental virus Asia1/JSp1c8 and recombinant viruses (FMDV-RSD and FMDV-RDD) in cattle and pigs. After inoculation, a number of disease parameters were analyzed, including fever, clinical score, and viremia. The animals, except for the FMDV-RSD-inoculated animals, showed fever and extensive tissue damage at the inoculation sites by day 1 and achieved the maximal score of lesions on day 2-4. Some FMDV-RSD-inoculated animals developed fever and tissue damage by day 2 and achieved the maximal score of lesions on day 3-5. Two animals (infected with FMDV-RSD) had no evidence of tissue damage, except for occasional depression and anorexia when their body temperatures rose. The Asia1/JSp1c8 and FMDV-RDD viruses produced more extensive tissue damage at the injected sites and induced fever and vesicles a day earlier than in the FMDV-RSD-inoculated animals. There were significant differences in lesion scores between RDD viruses (Asia1/JSp1c8 and FMDV-RDD) and RSD virus (P < 0.05, P < 0.05), however, no significant differences in lesion scores between cattle and pigs (P > 0.05). The lesion scores for the inoculated animals are summarized in table 3 and figure 3 shows the rectal temperature of all of the inoculated animals. The disease was characterized by viremia in all inoculated animals, including the animals that did not generate vesicular lesions. The level of viremia increased following inoculation, typically reaching a peak level after two or three days then decreasing to zero by day 8. In contrast to the lesion scores, there were no significant differences caused by viruses or species in terms of the maximum viremia (P = 0.56), time of maximum viremia (P = 0.75) or time of maximum rate of increase in viremia (P = 0.69). Virus RNA copies in the blood of the inoculated animals are summarized in table 4.

**Table 3:** Lesion scores of all animals on days-post inoculated

Virus	**Animal no**.	**Lesion scores of days-post inoculation**^***a***^							
		
		Day1	Day2	Day3	Day4	Day5	Day6	Day7	Day8
Asia1/JSp1c8	Bovine88	1	3	5	5	5	5	5	5
	Bovine91	1	3	3	4	4	4	4	4
	Pig451	1	4	5	5	5	5	5	5
	Pig453	1	1	2	4	4	4	4	4
	Pig454	1	3	5	5	5	5	5	5
FMDV-RDD	Bovine 96	1	3	4	5	5	5	5	5
	Bovine 99	1	3	5	5	5	5	5	5
	Pig 458	1	1	3	3	3	3	3	3
	Pig 459	1	2	4	5	5	5	5	5
	Pig 460	1	4	5	5	5	5	5	5
FMDV-RSD	Bovine 100	0	0	0	0	0	0	0	0
	Bovine 101	0	0	3	3	3	3	3	3
	Pig 461	0	1	3	3	4	4	4	4
	Pig 462	0	0	0	0	0	0	0	0
	Pig 465	0	2	3	3	3	3	3	3

^*a *^Lesion scores were calculated as described by Rieder *et al*. (2005).

**Table 4:** Virus RNA copies detected in the blood of all animals on days-post inoculated

Virus	**Animal no**.	**Virus RNA copies in the blood of days-post inoculation(× 10**^**6**^**)**^***b***^							
		
		Day1	Day2	Day3	Day4	Day5	Day6	Day7	Day8
Asia1/JSp1c8	Bovine88	0.1	14	4	0.9	2.6	1.1	0	0
	Bovine91	0.3	1.0	14.5	6	0.1	0	0	0
	Pig451	0.04	17	4.6	2.1	0.4	0	0	0
	Pig453	0.06	4	11.7	1	0.3	0	0	0
	Pig454	0.2	9	96.4	10	5	1.8	0.2	0
FMDV-RDD	Bovine 96	2	17.4	42.9	8.8	3.1	4.2	0	0
	Bovine 99	9	78.8	9.4	2.3	0.3	0	0	0
	Pig 458	0.03	0.6	22.5	5.5	3.9	1	0.2	0
	Pig 459	0.2	2.3	30.2	14.4	3.1	0.2	0	0
	Pig 460	0.3	2.8	36.9	15.1	2	0.3	0	0
FMDV-RSD	Bovine 100	0.02	0.2	7.8	3.8	2.1	0.2	0	0
	Bovine 101	0.1	3	12.6	16.2	9.8	6.2	2.3	0
	Pig 461	0.4	6.9	19.6	10.5	5.1	2.8	0.5	0
	Pig 462	0	0.1	14.6	7.1	1	0.9	0	0
	Pig 465	0.02	3.6	16.6	10.4	5.2	1.1	0.9	0

^*b *^The amount of virus in the blood was measured by real-time quantitative RT-PCR assay as described in materials and methods. Blood samples were collected at 1-8 dpi in inoculated animals.

Vesicular fluid was collected from inoculated animals, and each sample was separately processed for RT-PCR and nucleotide sequencing. The results revealed that the original receptor-binding motif did not change during growth in vivo.

## Discussion

The RGD integrin-binding motif within VP1 is highly conserved among FMDV field isolates, and is generally considered essential for virus viability via its interaction with cellular integrin heterodimers [24-26]. Biochemical evidence that small peptides containing the RGD sequence inhibited the adsorption of the virus to tissue culture cells [11], and genetically engineered virions containing either mutations or deletions of the RGD sequence were unable to bind to cells or cause disease in susceptible animals [12,25,27]. However, the RGD triplet may be dispensable upon short-term evolution of the virus harboring it in a constant environment [21,28,29]. In the present report, we have documented that non-RGD derivatives arose serendipitously during short-term passage of a FMDV field isolate in vitro and in vivo. One derivative containing an RDD triplet in the receptor-binding site was obtained from the serotype Asia 1 field isolate after a single cattle-to-pig transmission and subsequent BHK-2**1 **in vitro passage. Sequence analysis of 10 biological clones of the VP1 encoding region of this population demonstrated that RDD viruses instead of the original RGD virus became predominant at an early phase of Asia1/JS/CHA/05 quasispecies evolution. Unexpectedly, however, both RGD and RSD viruses were obtained from the Asia1/JSM4 population that were generated after four serial passages of the Asia1/JS/CHA/05 field isolate in suckling mice, via intraperitoneal inoculation. The population equilibrium of RSD mutant and ancestor viruses was maintained after 20 passages of the Asia1/JSM6 population in BHK-21 cells. Although RDD- or RSD-containing FMDV are unusual, they were genetically stable upon extended replication in cell culture. Our results suggest that, in the context of the capsid proteins of Asia1/JS/CHA/05, a highly conserved RGD motif is not essential for replication in vitro and in vivo, suggesting functional flexibility of FMDV to enter cells in response to environmental modifications.

Like other RNA viruses, FMDV exists as closely related but non-identical genomes, termed viral quasispecies [30,31]. Genetic diversity is an intrinsic property of the quasispecies, which arise due to the lack of proofreading activity during viral genome replication, a short replication cycle, and other environmental selective pressures [32,33]. Our observations showed that evolution of FMDV population exhibited receptor binding motif diversity (genetic diversity) subjected to short-term passage of field isolate in different environments. From the standpoint of RNA virus population evolution, one possible scenario could explain this observation. The early interactions between viruses and host cells exert major selective force on virus populations, thus, the variants (RSD- and RDD-containing viruses) may already be present at low frequency in the natural population that are possibly more fit in new environments and become dominant strains. While this presumption is contrary to the view that the RGD triplet is highly conserved among natural isolates of FMDV, there is direct evidence that an RDD containing field virus was isolated from pigs during a type Asia 1 FMD outbreak in China. RDD-containing FMDV VP1 genes were amplified from sheep oesophageal-pharyngeal fluids (OP-fluids) collected during 2006 from a sheep herd in the region of China that had endemic Asia 1 serotype FMDV [34,35]. The emergence of these non-RGD mutants in nature is likely to be influenced by specific epidemiological and immunological aspects of host-virus interaction as well as the quasispecies composition of the viral population [36-39].

The molecular basis for this apparent relaxation of the requirement for the precise RGD sequence is not well understood, but functional replacements within the RGD triplet allow the use of an alternative receptor for entry of FMDV in BHK-21 cells. Since other FMDV lack the RGD motif, host cell recognition may be mediated through another integrin receptor or a non-integrin pathway, or use a third receptor (neither integrin-based nor HS) for entry into the host cell [18,21,40]. Further studies are required to analyze the interaction of these mutants with the major FMDV integrin receptors αvβ3, αvβ6, αvβ1 and αvβ8 identified to date, and to understand whether these viruses obtain alteration of cell tropism, antigenicity, and virulence.

To examine the influence of single amino acid substitutions in the receptor binding site of RDD-containing FMD viral genome on virus viability and the ability of non-RGD viruses to cause disease in susceptible animals, we constructed an FMDV Asia1/JS/p1c8 full-length clone and derived mutant molecules with RGD or RSD receptor recognition motifs. Following transfection of BSR cells with these clones, three recombinant viruses were rescued, in particular, six other amino acid differences in the P1 capsid region of Asia1/JS/CHA/05 and Asia1/JSM4 (compared with Asia1/JS/p1c8) did not affect rescue of viable RGD- and RSD-harboring viruses. Furthermore, in vitro growth properties of these viruses did not differ significantly. Our results showed that Asia1/JS/p1c8 viral genome can tolerate substitutions in the receptor binding site with no other changes in the capsid. The ability of the Asia1/JS/p1c8 viral genome to tolerate substitution of receptor binding sites may depend on the capsid sequence, because the Asp-143 Gly change of receptor recognition site was lethal in the context of the capsid proteins of FMDV C-S8c1. However, the same replacement yielded viable viruses in the context of the capsid protein of FMDV C-S8c1p100 and C-S8c1p213 [21,41].

To assess the ability of non-RGD FMD viruses to cause disease in naturally susceptible animals, we performed experiment infections of cattle and pigs using the Asia1/JS/p1c8 and two non-RGD recombinant viruses. Subsequent experiments showed that all viruses were able to cause disease in cattle and pigs and produce rapid onset of clinical signs, characteristic of infection with RGD field strains. The disease was characterized by viremia in all inoculated animals, including the individuals that did not generate vesicular lesions. Amongst these viruses, the RSD virus produced less tissue damage at the inoculation sites and induced fever and vesicles a day later than in the animals inoculated with RDD-containing viruses, which indicated a different degree of disease severity. The different virulence of these viruses was also supported by the maintenance of original receptor recognition sequence in vesicle samples obtained from infected animals. The reason for low virulence of RSD virus, which may bear on receptor usage, needs to be further explored. These observations are particularly interesting, since the presence of an RGD motif is believed to be the main determinant to direct FMDV to integrin-containing target tissues during infection in the natural host [42]. In addition, information currently available indicates that FMDV utilizes integrins for entry in the natural host, and there is no evidence of the use of alternative receptors in vivo [5,14,28]. Therefore, our results further support the possibility that a non-RGD-integrin interaction could be responsible for the generation of FMD in the natural host. Our study was the first to demonstrate the ability of an RDD containing natural isolate to cause disease in naturally susceptible animals, and will provide knowledge about the in vivo pathogenesis of non-RGD viruses.

## Conclusion

FMDV quasispecies evolving in a different biological environment gained the capability of selecting different receptor recognition sites. Thus, the early interaction between the viruses and the host cells may exert major selective pressure on FMDV populations that contributes to the evolution and functional flexibility of FMDV to enter cells. Our studies using two non-RGD FMDVs not only show that there was an increase in the number of viable mutants with substitutions in the receptor-binding region, but also provide useful tools for studies of cell recognition by FMDV. Based on an RDD-containing full-length infectious cDNA clone, the RSD- and RGD-containing recombinant viruses were rescued, and single amino acid substitutions in the receptor-binding site did not affect virus viability. The viruses expressing non-RGD receptor binding sites can replicate stably in vitro and induce the disease in susceptible animals.

## Methods

### Viruses and cells

**Figure 4:**
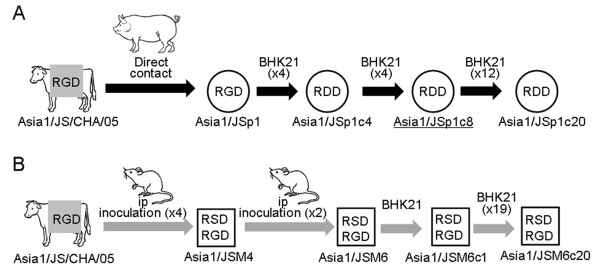
**Passage history and origin of FMDVs used in this study derived from a field isolate, Asia1/JS/CHA/05**. The Asia1/JSp1c8 and Asia1/JSM4 population with alternative RGD motifs were occasionally found by two different passage strategies (**A **and **B**). Nomenclature used for the passaged viruses is as follows: "p" denotes passage number in pig; "M" denotes passage number in suckling mice and "c" denotes passage number in BHK-21 cells. The virus used for construction of full-length infectious cDNA clone is underlined.

FMDV Asia1/JS/CHA/05 utilized in this study was originally isolated from cattle in Wuxi, Jiangsu Province, China, in 2005. The complete genome sequence of this virus was published in GenBank (GenBank Accession: EF149009). FMDV Asia1/JSp1c8 is a viral population resulting from eight serial passages of Asia1/JSp1 virus in BHK-21 cells, as previously described [43], which was obtained from a pig infected by placing it in contact with an Asia1/JS/CHA/05 virus-inoculated cattle. FMDV Asia1/JSM4 is a viral population resulting from four serial passages of Asia1/JS/CHA/05 virus in suckling mice, via intraperitoneal inoculation. Figure 4 shows the passage history of Asia1/JS/CHA/05 field isolate in different environments.

BSR-T7/5 cells (a cell line derived from BHK-21, which constitutively expresses T7 RNA polymerase [44]) were maintained in Glasgow minimal essential medium (GMEM) supplemented with 4% tryptose phosphate broth, 10% fetal bovine serum (FBS) and were additionally provided with G418 (1 mg mL^-1^) on every second passage to ensure maintenance of the T7 polymerase gene. BHK-21 cells were grown in Eagle's minimal essential medium (EMEM) supplemented with 10% FBS.

### RNA extraction, RT-PCR and nucleotide sequencing

RNA was extracted from virus stock of Asia1/JSp1c8, Asia1/JSM4, and Asia1/JS/China/2005 using RNeasy mini kit (Qiagen, Valencia, CA) according to the manufacturer's instructions. Viral cDNAs were synthesized from the viral RNAs, as previously described [45]. Briefly, viral cDNAs were synthesized using M-MLV reverse transcriptase (Invitrogen, Carlsbad, CA, USA) with NK61 primer (5'-GACATGTCCTCCTGCATCTG-3') and the VP1 coding regions were amplified by PCRs with the primer pair NK61/VP31 (5'-TAGTGCTGGYAARGACTTTG-3'). The PCRs were performed using PrimeSTAR HS DNA Polymerase (Takara, Dalian, China). PCR amplifications were carried out for 30 cycles of denaturation at 98°C for 20 s, annealing at 68°C for 1 min, and extension at 72°C for 8 min. Following amplification, the cDNA fragments were purified from agarose gels using a kit (Qiagen) and sequenced by Sunny Biotech (Shanghai, China). In order to detect heterogeneity of the VP1 gene, the amplicons were cloned into a pGEM-T vector (Promega, Madison, WI, USA) using standard molecular cloning techniques [46] and plasmids derived from 10 positive clones for each sample were sequenced. Additionally, the capsid-encoding regions of Asia1/JSp1c8, Asia1/JSM4, and Asia1/JS/CHA/05 were also amplified and sequenced.

### Construction of genome-length cDNA clone of Asia1/JSp1c8 and derivation of G-H loop VP1 mutants

**Figure 5:**
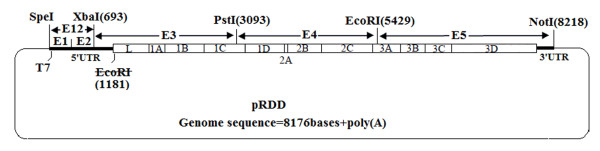
**Strategy used to construct FMDV Asia1/JSp1c8 full-length cDNA clone, pRDD**. The location of restriction enzyme cleavage sites used to assemble the subcloned RT-PCR fragments (E1, E2, E3, E4, E5 and E12) are shown (numbered relative to nucleotide position in the virus genome). Thick lines and an open box represent the untranslated regions and the open-reading frame for the viral polyprotein, respectively. The thin line represents the vector sequence. FMDV cDNA is under the control of the T7 promoter.

Recombinant DNA techniques were used according to standard procedures [46]. The viral RNA of Asia1/JSp1c8 was used as a template for first-strand cDNA synthesis with M-MLV reverse transcriptase by using specific oligonucleotide primers (E1', E2', E3', E4', and E5'). A total of five fragments (E1-E5; Figure 5), covering the complete virus genome, were subsequently amplified by PCR. Two fragments (E1 and E2 corresponding to nucleotide 1-390, 362-700) were amplified with the E1/E1' and E2/E2' primer pairs by PCR. T7 RNA polymerase promoter was introduced in the E1 primer. Cycling conditions for both PCRs were as follows: initial denaturation at 94°C for 1 min, 30 cycles of 98°C for 20 s, 68°C for 40 s, and then 72°C for 8 min. E12 fragments were generated by overlap PCR fusion E1 and E2 fragments with primer pair E1/E2'. PCR amplifications involved initial denaturation at 94°C for 1 min, followed by 30 cycles of 98°C for 20 s, 68°C for 1 min, then 72°C for 8 min. The amplicons were ligated into the pGEM-T vector, leading to the positive clone pGEME12. The other three fragments (E3, E4 and E5 corresponding to nucleotide 690-3101, 3090-5437 and 5425 to the 3'-end) were produced by PCR with primer pairs E3/E3', E4/E4', E5/E5'. Cycling parameters for three PCRs were as follows: initial denaturation at 94°C for 1 min, 30 cycles of 98°C for 20 s, 68°C for 3 min, and then 72°C for 10 min. The E3, E4 and E5 amplicons were cloned into the M-pSK vector with *XbaI/PstI*, *PstI/EcoRI*, and *EcoRI/NotI *sites, the resulting positive plasmids were designated pSKE3, pSKE4, and pSKE5, respectively. The M-pSK vector derived from pBluescriptSK (+) by removed T7 promoter and modified some restriction enzyme sites in the vector sequence, was synthesized by GenScript Biotech Company (Nanjing, China). To introduce the genetic tags into the genome of Asia1/JSp1c8, recombinant plasmid pSKE3Δ, which contained two synonymous mutations (1185A→G, 1185T→C) to eliminate the *EcoR*I site in the E3 fragment, were constructed by oligonucleotide-directed mutagenesis with PCR amplification of the parent plasmid pSKE3 using p1/p1'primer pair. PCR amplification was carried out for 18 cycles of denaturation at 95°C for 30 s, annealing at 55°C for 1 min, and extension at 68°C for 8 min. All recombinant plasmids were confirmed by complete DNA sequencing. Primers used to construct full-length cDNA clones of Asia1/JSp1c8 are listed in table 5.

**Table 5:** Sequences of the primers used for the construction of a full-length cDNA clone and mutants of FMDV Asia1/JSp1c8

Name	Nucleotide Sequence (5'→3')	Nucleotide Position (nt)
E1	CAGGATCC*TAATACGACTCACTATAGGG*TTGAAAAGG GGCGCTAGGGTC	1-21
E1'	TAAAACTTAGGGGGGGGGGGGGGGGGGGGTGAAAG	361-390
E2	TTTCACCCCCCCCCCCCCCCCCCCCTAAGTTTTAC	362-391
E2'	CCTCTAGA CCTGGAAAGACCAGGC	677-700
E3	AGGTCTAGAGGGGTGACATTTTGT	690-713
E3'	GTCTGCAGCAGAAAGGTAAGGGAT	3078-3101
E4	CTGCTGCAGACTATGCTTACACTG	3090-3113
E4'	AAAGAATTC AATTGCTGCCTCATG	5414-5437
E5	AATTGAATTCTTTGAGGGAATGGTGCAC	5425-5452
E5'	TTGCGGCCGCTTT(38)	3'end
P1	ACAAGGAAAGATGGAGCTCACACTTCACAAC	1168-1198
P1'	GTTGTGAAGTGTGAGCTCCATCTTTCCTTGT	1168-1198
TR1	ACTGCATTCATTCTGAGTGGGA	2960-2984
TR1'	GGCAAGATCACCACGCCGCGAGGA	3679-3703(D→G)
TR2	TCCTCGCGGCGTGGTGATCTTGCC	3679-3703(D→G)
TR2'	5'-GAAGAAACTCGAGGCGACTTTGAC-3'	4342-4366
TR3	TCCTCGCGGCGTAGTGATCTTGCC	3679-3703(D→S)
TR3'	GGCAAGATCACTACGCCGCGAGGA	3679-3703(D→S)

Nucleotide positions of primers used for cloning are shown: numbering according to Asia1/JS/CHA/05 (Genbank Accession: EF149009). T7 promoter sequences are marked with italic type, the restriction sites are highlighted by underlining, silent mutations within the viral sequence are underlined and amino acid changes are shown in brackets.

The full-length virus genome was assembled by a series of ligation steps (Figure 5). First, a 2400-bp *XbaI*-*PstI *fragment was release from plasmid pSKE3Δ and cloned into plasmid pGEME12 digested with *PstI *and *XbaI*, leading to the construct pGEME123. A 3123-bp *SpeI*-*PstI *fragment of the pGEME123 was inserted into the pSKE4 plasmid digested with *SpeI *and *PstI*, the resulting plasmid pSKE1234. A 5429-bp *SpeI*-*EcoRI *fragment was release from plasmid pSKE1234 and ligated into plasmid pSKE5 digested with *EcoRI *and *SpeI*, the resulting plasmid named pRDD, which contained genome-length cDNA clone of Asia1/JSp1c8, was sequenced to confirm sequence fidelity.

Overlapping PCRs were used to introduce amino acid substitutions (144 D (gat) to G (ggt), 144 D (gat) to S (agt)) into the structural protein VP1 of Asia1/JSp1c8 virus. Individual parts were amplified with primer pairs TR1/TR1', TR2/TR2', TR1/TR3' and TR3/TR2' (Table 5), and then both overlapping PCR fusion reactions were performed by mixing PCR-amplified fragments with TR1/TR2' primer pair. The parameters of two PCRs as following: initial denaturation at 94°C for 1 min, 30 cycles of 98°C for 20 s, 68°C for 1 min, and then 72°C for 8 min. The two fused PCR fragments were digested with *EcoR*I and *Sac*II and cloned into the full-length plasmid pRDD. The mutated full-length cDNA clones named pRGD, and pRSD, respectively, were sequenced through the entire amplified regions to confirm the presence of the expected modifications.

### Virus rescue

The plasmids pRDD, pRGD and pRSD were linearized with *No*tI and purified from agarose gels with columns (Qiagen). BSR-T7/5 cells (4-6 × 10^5 ^in a six-well plate) were transfected with mixtures containing 2 μg each of three linearized plasmids and 10 μL Lipofectamine 2000 (Invitrogen) according to the manufacturer's directions. As a negative control, Lipofectamine 2000 was also used to transfect BSR-T7/5 cells. After 6 h of incubation at 37°C, the cells were added to GMEM supplemented with 10% FBS and further incubated for 72 h at 37°C with 5% CO_2_. The cell culture supernatants were harvested at 72 h post-transfection and were then serially passaged 10 times on BHK-21 cells to increase virus titers.

### Replication kinetics of rescued FMDVs

Growth kinetics of the viruses was determined in BHK-21 cells. Confluent monolayers in 60 mm diameter plates were infected at a multiplicity of infection (MOI) of 10 PFU per cell with Asia1/JSp1c8 virus and the three genetically engineered viruses. After adsorption for 1 h, the monolayers were washed with 0.01 M phosphate-buffered saline (PBS; pH7.4), and maintained in DMEM supplemented with 2% FBS at 37°C with 5% CO_2_. The virus-infected supernatants were collected at 4, 8, 12, 16 and 24 h after inoculation. Plaque assays were performed using gum tragacanth overlay and crystal violet staining, as previously described [47]. Virus titers (plaque-forming units (pfu) mL^-1^) were determined on BHK-21, as described elsewhere [48].

### Animal experiments

Nine 2-month-old pigs and six 1-year-old bovines were divided into three groups, each consisting of three pigs and two bovines. All animals were seronegative for FMDV non-structural protein (NSP) antibodies prior to experimental infection. Two non-RGD recombinant viruses and Asia1/JSp1c8 virus with a titer of 1.6 × 10^7 ^pfu mL^-1^, 1.3 × 10^7 ^pfu mL^-1^, and 1.0 × 10^7 ^pfu mL^-1^, respectively, were used to separately inoculate animals. Each pig was inoculated with 2 mL inoculum via the intramuscular route, each bovine received 1 mL intramuscularly and 1 mL via the tongue. Following inoculation, animals were carefully scored for appearance of lesions at inoculation sites and at other sites. Lesion scores were based on the number of sites affected that were distinct from actual injection sites. Scores were calculated as described by Rieder et al [28].

The viral load in the blood was assessed by real-time quantitative RT-PCR using the RNA Master SYBR green I kit (Roche), as specified by the manufacturer. Quantification was relative to a standard curve obtained with known amounts of FMDV O/CHA/99 RNA, using a procedure that has been described previously [49], except that the primers (patent pending) targeted the 3D non-structural protein were altered. The viral RNA was extracted from vesicular fluid (collected on selected days), reverse transcribed, and sequenced through the entire VP1 region as described above.

All animal studies were approved by the Review Board of Lanzhou Veterinary Research Institute, Chinese Academy of Agricultural Sciences (Permission number: SYXK-GAN-2004-0005). All animals used in this study were humanely bred during the experiment and euthanasia was carried out at the end of the experiment to reduce suffering.

### Statistical analysis

Changes in viral titer over time for the in vitro passage experiments were modeled using linear models with virus and time since infection (treated as a factor) as fixed effects. Model selection proceeded by stepwise deletion of non-significant terms (as judged by *F*-tests) starting from a model including virus, time since infection and an interaction between these factors.

Lesion scores over time were modeled using linear mixed models with virus and species as fixed effects and animal identification number as a random effect. Model selection proceeded by stepwise deletion of non-significant terms (as judged by the Akaike information criterion; AIC) starting from a model including virus, species and an interaction between these factors.

The change in viremia (y, measured as viral RNA copy number) over time (*t*) was described using the following function,

where α is the maximum viremia, is the time at which maximum viremia occurs and is the time before (after) maximum viremia at which the maximum rate of increase (decrease) in viremia occurs. Parameters for each animal were estimated by fitting the curve to the data using the method of least-squares. Estimates for each animal were compared using Kruskal-Wallis tests to identify significant differences (P < 0.05) amongst animals infected with different viruses. This analysis was done for all animals and then repeated for cattle only and for swine only. Animal B99 was excluded from the analysis of viremia because robust estimates could not be obtained for the parameters.

## Competing interests

The authors declare that they have no competing interests.

## Authors' contributions

PHL and ZJL conceived and designed the study. PHL and WJC constructed three FMDV full-length infectious cDNA clones. DL and XWB carried out the animal experiments. HFB and PS carried out the real-time quantitative RT-PCR assay. HY and ZXL supervised all aspects of the research. YLC, BXX and JHG passaged the three recombinant viruses respectively. PHL and DPK co-drafted the manuscript. SG aligned the data and conducted statistical analysis. All authors read and approved the final manuscript.
